# Novel insights into the m^6^A-RNA methyltransferase METTL3 in cancer

**DOI:** 10.1186/s40364-021-00278-9

**Published:** 2021-04-20

**Authors:** Yiqing Cai, Rui Feng, Tiange Lu, Xiaomin Chen, Xiangxiang Zhou, Xin Wang

**Affiliations:** 1 Department of Hematology, Shandong Provincial Hospital, Cheeloo College of Medicine, Shandong University, No.324, Jingwu Road, Jinan, 250021 Shandong China; 2 Department of Hematology, Shandong Provincial Hospital Affiliated to Shandong First Medical University, Jinan, 250021 Shandong China; 3 School of Medicine, Shandong University, Jinan, 250012 Shandong China; 4 Shandong Provincial Engineering Research Center of Lymphoma, Jinan, 250021 Shandong China; 5 Branch of National Clinical Research Center for Hematologic Diseases, Jinan, 250021 Shandong China; 6 National Clinical Research Center for Hematologic Diseases, The First Affiliated Hospital of Soochow University, Suzhou, 251006 China

**Keywords:** N6-methyladenosine, METTL3, RNA regulation, Tumorigenesis

## Abstract

N6-methyladenosine (m^6^A) is a prevalent internal RNA modification in higher eukaryotic cells. As the pivotal m^6^A regulator, RNA methyltransferase-like 3 (METTL3) is responsible for methyl group transfer in the progression of m^6^A modification. This epigenetic regulation contributes to the structure and functional regulation of RNA and further promotes tumorigenesis and tumor progression. Accumulating evidence has illustrated the pivotal roles of METTL3 in a variety of human cancers. Here, we systemically summarize the interaction between METTL3 and RNAs, and illustrate the multiple functions of METTL3 in human cancer. METLL3 is aberrantly expressed in a variety of tumors. Elevation of METTL3 is usually associated with rapid progression and poor prognosis of tumors. On the other hand, METTL3 may also function as a tumor suppressor in several cancers. Based on the tumor-promoting effect of METTL3, the possibility of applying METTL3 inhibitors is further discussed, which is expected to provide novel insights into antitumor therapy.

## Introduction

Epigenetics promotes the functional plasticity of genome at multiple levels [1]. As the classical kinds of chemical modifications, 5-methylcytidine (m^5^C), 5-hydroxymethylcytidine (hm^5^C), N4-acetylcytidine (ac^4^C), and N6-methyladenosine (m^6^A) mainly participate in the epigenetic modification of RNAs [2]. Among the different kinds of modifications, m^6^A is the most common and effective modification in both coding and noncoding RNAs [3, 4]. The importance of m^6^A modification has been recognized in physiological and pathological processes [5–7]. Meanwhile, m^6^A modification also plays critical roles in yeast and plants [8, 9].

The dynamic progress of m^6^A modification is driven by the interactions between “writers”, “erasers”, and “readers” [10, 11]. The m^6^A deposition is primarily performed by “writers”, while the modification site is subsequently “read” by m^6^A recognition proteins or “erased” by m^6^A demethylases [12]. In particular, human N6-methyltransferase complex (MTC), which contains Methyltransferase-like 3 (METTL3) [13], METTL14 [14], Wilms tumor 1-associated protein (WTAP) [15], METTL16 [16], KIAA1429 [17], zinc finger CCCH-type containing 13 (ZC3H13) [18], RNA-binding motif protein 15 (RBM15) [19], and Cbl proto-oncogene like 1 (CBLL1) [20], is responsible for methyl group transfer. As the core component of MTC [21], METTL3 dominates the catalytic core and performs N6-methylase catalytic activity [22]. Dysregulation of METTL3 significantly affects the total m^6^A methylation level [23]. In addition, noncatalytic components of the complex also contribute to the RNA methylation progression. METTL14 assists to construct the RNA binding scaffold to promote the RNA binding ability of METTL3, thereby enhancing the catalytic effect of METTL3 [24]. In addition, WTAP can facilitate the nuclear speckle localization of METTL3 and METTL14 [24]. Apart from the essential components, recent studies have demonstrated that METTL16, KIAA1429, ZC3H13, RBM15 and HAKAI are involved in m^6^A modification in various ways [12].

**Fig. 1:**
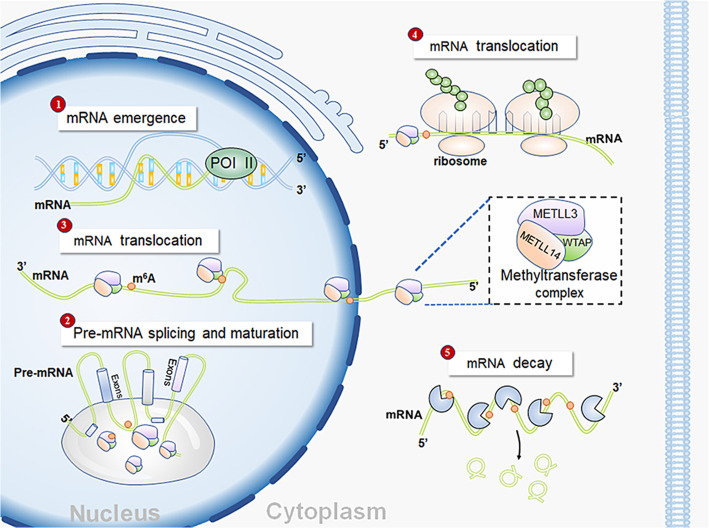
In the nucleus, METTL3 promotes mRNA splicing through recognizing the 3’UTR m^6^A sites on mRNA, thereby altering the mRNA structure. Moreover, METTL3 also transports from nucleus to the cytoplasm and further enhances the translation and degradation of mRNA

It has been well recognized that RNA methylation influences the metabolic processes and functional regulation of RNA. As the critical component of MTC, METTL3 primarily affects post-transcriptional genetic modification (Fig. 1). Genetic modification leads to changes in biological processes, including cell growth, migration, differentiation and inflammatory response [25]. Recently, increasing studies have revealed the accumulation of m^6^A modification in human cancers, indicating the important role of METTL3 in tumorigenesis and tumor progression [26]. In this review, we systematically summarize the functions of METTL3 in different human malignancies and further discuss the potential of METTL3 inhibitors.

## Reciprocal effects between METTL3 and RNAs in human cancers

Methyltransferase activity of METTL3 can be detected both in the nucleus and cytoplasm [27], suggesting that METTL3 could modulate the metabolism and function of RNAs in various ways. On the other hand, expression and functions of METTL3 can also be regulated by noncoding RNAs.

### METTL3 regulates the maturation, transportation and translation of messenger RNA (mRNA)

METTL3 was involved in the regulation of mRNA, including the maturation, transportation and translation of mRNA [25]. Nucleus-localized METTL3 primarily promoted the maturation, splicing and transportation of mRNA. The abundant deposition of m^6^A in pre-mRNA was associated with the acceleration of pre-mRNA maturation [27]. After pre-mRNA production, methylation of spliced regions could affect the splicing of the pre-mRNA, thereby producing diverse sequences of mature mRNA [28]. In addition, increasing m^6^A on mature mRNA decreased the nuclear fraction of mRNA by promoting cytoplasmic transportation [17]. In the METTL3-enriched cytoplasm, METTL3 significantly enhanced mRNA translation by stabilizing mRNA [29]. Mechanistically, m^6^A modification frequently took place in the coding sequence (CDS), the 3′UTR and regions near the stop codons of mature mRNA [24]. Recognition proteins specifically recognized the m^6^A-abundant regions to stabilize mRNA and further enhance translation of mRNA in an m^6^A-dependent manner [24]. In addition, the interplay between specific transcription factor and METTL3 could also promote translation. For instance, functional interaction between METTL3 and eukaryotic translation initiation factor 3 subunit h (eIF3h) was required for enhanced translation and oncogenic transformation [30]. Apart from its promoting effect, METTL3 could also increase mRNA decay [31], revealing the dual effects of METTL3 on mature mRNA.

### METTL3 promotes the maturation and activation of noncoding RNAs

m^6^A modification can regulate the maturation, transport, stability and degradation of noncoding RNAs, eventually affecting the biological function of tumor cells [32]. METTL3 was proved to promote the maturation and activation of microRNA (miRNA). m^6^A modification on the primary miRNA (pri-miRNA) directly facilitated the maturation of pri-miRNA, consequently promoting tumor progression [33]. For example, METTL3 modulated pri-miR221/222 maturation in an m^6^A-dependent manner in bladder cancer (BC). Mature miR221/222 then suppressed the expression of phosphate and tension homology deleted on chromosome 10 (PTEN) to accelerate tumor growth [34]. In addition, mature miR-1246 induced by METTL3 could activate the mitogen-activated protein kinase (MAPK) pathway by suppressing sprouty-related EVH1 domain protein 2 (SPRED2), which facilitated the invasion and distant metastasis of colorectal cancer (CRC) [35]. On the other hand, METTL3 could indirectly promoted miRNA activation by regulating long noncoding RNA (lncRNA). The activation of miR-1914-3p induced by hypermethylated lncMALAT1 distinctly enhanced the expression of YAP, leading to rapid progression and enhanced therapeutic resistance of non-small cell lung cancer (NSCLC) [36].

### Noncoding RNAs regulate the expression and functions of METTL3

Noncoding RNAs, including miRNA and lncRNA, are involved in tumor progression by regulating METTL3. miRNA, which was regarded as specific transcription factors of METTL3, could decrease the expression and function of METTL3, thereby reversing the tumor-promoting effect of METTL3 [37–39]. lncRNA also participates in the regulation of METTL3. For example, the interaction between LINC00470 and METTL3 facilitated the degradation of PTEN mRNA and further contributed to the development and progression of gastric cancer (GC) [40]. In addition, lncRNA Rho GTPase activating protein 5 (ARHGAP5)-AS1 recruited METTL3 to enhance the stability of ARHGAP5 mRNA, eventually leading to the poor prognosis and chemoresistance of GC [41].

## METTL3 mediates tumorigenesis via RNA methylation

An increasing number of studies have illustrated that METTL3 is involved in various aspects of tumor progression, including stemness maintenance, tumor growth, invasion, migration, and drug resistance.

### Gastrointestinal tumors

#### GC

Aggressive GC is generally accompanied with higher expression of METTL3, suggesting the oncogenic role of METTL3 in GC [42]. Elevation of METTL3 was proved to promote tumor growth, metastasis and therapeutic resistance in an m^6^A-dependent pattern [43]. METTL3 propelled tumor growth by inducing m^6^A deposition on the mRNA of oncogenes and rate-limiting enzymes of glucose metabolism. MYC and SEC62 were previously identified as oncogenes in GC. Abundant m^6^A deposition was not only detected on the component molecules of the MYC-targeted genes, but also contributed to the overexpression of MYC [44, 45]. Another study demonstrated that METTL3 promoted the stability of SEC62 mRNA in an m^6^A-mediated manner and further inhibited tumor cell apoptosis by suppressing the Bax-caspase3 pathway [46]. Apart from oncogenes, aerobic glycolysis was also activated in tumorigenesis [47]. Mechanistically, m^6^A modification enhanced the expression of hepatoma-derived growth factor (HDGF), which could activate solute carrier family 2 member 4 (GLUT4) and enolase 2 (ENO2) to potentiate aerobic glycolysis in tumor cells [48]. Moreover, the interaction between METTL3 and lncRNA LINC00470 promoted tumor growth by impairing the stability of PTEN mRNA [40].

Tumor metastasis and therapeutic resistance represent the characteristics of aggressive GC [43]. Epithelial mesenchymal transition (EMT) and angiogenesis provide proper conditions for cell mobility. Overexpression of zinc finger MYM-type containing 1 (ZMYM1) was induced by METTL3, thereby promoting the EMT process by suppressing the activation of E-cadherin [49]. Meanwhile, expression levels of EMT-related markers, especially growth factor independent 1 (GFI-1) and α-smooth muscle actin (α-SMA), were dramatically increased under the regulation of METTL3 [43]. The angiogenesis process, especially the proliferation of human umbilical vein endothelial cell (HUVEC) and tube formation, was correlated with overexpressed METTL3, subsequently promoting the invasion and distant migration of cancer cells [48]. The contribution of METTL3 to chemoresistance was verified as well. Mechanistically, METTL3 contributed to the stabilization of ARHGAP5 mRNA after being recruited by lncRNA ARHGAP5-AS1 and then induced chemoresistance [41].

On the other hand, METTL3 can suppress tumor progression under certain conditions. Xie et al. reported that METTL3 facilitated the m^6^A modification on basic leucine zipper ATF-like transcription factor 2 (BATF2) mRNA. Methylated BATF2 exerted tumor suppressive effects by stabilizing the p53 protein and inhibiting the phosphorylation of extracellular regulated kinase (ERK) [50]. In addition, METTL3-high GC cells preferred to respond to rapamycin (mTOR) inhibitors through m^6^A-DGCR8-dependent mechanism [51].

#### Hepatocellular cancer (HCC) and gallbladder cancer

Increased METTL3 is not only involved in tumorigenesis but is also related to rapid progression and poor prognosis of HCC [52, 53]. Mechanistically, METTL3 facilitated tumor progression by modulating suppressor of cytokine signaling 2 (SOCS2) [54], RAD52 motif 1 (RDM1) [55] and Snail [56]. Depending on the m^6^A modification, SOCS2 mRNA was functionally silenced, thereby promoting the proliferation, migration and stemness maintenance of HCC cells [54]. Hypermethylation of RDM1 induced by METTL3 suppressed the expression of RDM1, leading to the activation of the Ras/Raf/ERK pathway in tumor progression [55]. In addition, METTL3 accelerated the accumulation of Snail, which was essential for the persistence of oncogenic properties [56]. On the other hand, METTL3 also functioned on oncogenic noncoding RNAs [57]. Elevation of METTL3 contributed to enhance the expression of miR-6079 and lncRNA LINC00958, thereby potentiating aerobic glycolysis [57, 58]. Chemosensitivity of HCC cells modulated by METTL3 was also reported recently. Mechanically, METTL3 enhanced the stability of forkhead box O3 (FOXO3) mRNA in a METTL3-m^6^A-YTHDF1-dependent manner, subsequently promoting sorafenib sensitivity and inhibiting angiogenesis and autophagy-associated pathways [59].

Consistent with HCC, the tumor-promoting effect of METTL3 has been recently revealed in gallbladder cancer. METTL3 functionally reduced the protein level of PTEN via m^6^A-induced maturation of miR-92b-3p and subsequently activated the phosphatidylinositol 3′-kinase (PI3K)/protein kinase B (AKT) pathway [60].

#### CRC

Aberrant expression of METTL3 is considered to be a frequent event in the development of CRC, in which METTL3 mediates tumorigenesis by regulating target genes and pathways in an m^6^A-dependent manner. Li et al. reported that methylation of SRY-box 2 (SOX2) mRNA effectively prevented SOX2 mRNA from degradation, thereby provoking self-renewal, proliferation and migration of CRC cells [61]. In addition, upregulated cyclin E1 [62], activated miR-1246-SPRED2-MAPK axis [35] as well as inhibited SOCS2 [63] and yippee-like 5 (YPEL5) [31] were also induced by METTL3-catalyzed m^6^A modification. Rate-limiting enzymes of aerobic glycolysis were regulated by METTL3 as well. Overexpression of hexokinase 2 (HK2) and GLUT1 was attributed to the METTL3-m^6^A-IGF2BP2/3-dependent mechanism and further accelerated glycolysis to accelerate tumor growth [64]. Apart from the tumor-promoting effect, several studies had demonstrated the tumor suppressor role of METTL3 in cell migration, implying the role as a double-edged sword of METTL3 in CRC [65]. Moreover, METTL3 had dual effects on therapeutic resistance as well. Hypermethylation distinctly enhanced the general protein level of the p53 R273H mutant and leucine-rich repeat containing G protein-coupled receptor 5 (LGR5), thereby enhancing drug resistance [66, 67]. On the contrary, depletion of METTL3/14 strengthened the sensitivity to anti-PD-1 treatment through activating the interferon-γ (IFN-γ)/signal transducer and activator of transcription 1 (STAT1)/interferon regulatory factor 1 (IRF1) pathway [68].

#### Pancreatic cancer (PC)

Accumulated studies have identified METTL3 as an independent prognostic factor for PC [69]. Elevated expression of METTL3 enhanced tumor growth and metastasis by promoting maturation of miR-25-3 and activation of the PI3K/AKT pathway [70]. Meanwhile, hypermethylation contributed to chemo- and radio-resistance dependent on the dysregulation of MAPK cascades, ubiquitin modification and RNA process regulation [71]. Functional enrichment analysis further demonstrated that METTL3 could participate in the epinephrine stimulus response and neutrophil-mediated immune reaction, but the underlying mechanisms remain to be further studied [72].

### Respiratory tumors

#### Nasopharyngeal cancer (NPC) and oral squamous cell carcinoma (OSCC)

It is well known that higher METTL3 is associated with advanced stage and distant metastasis, indicating the tumor-promoting role of METTL3 in NPC [73, 74]. METTL3 was reported to promote tumor growth and metastasis through functional regulation of NPC related genes. Zinc finger protein 750 (ZNF750) and enhancer of zeste homolog 2 (EZH2), which were identified as the tumor suppressor in NPC, could inhibit the growth and metastasis of NPC cells [75]. METTL3 contributed to the m^6^A modification of ZNF750 and consequently restrained cell apoptosis by inhibiting ZNF750/fibroblast growth factor 14 (FGF14) signaling [76]. METTL3 also inhibited the translation of EZH2, thereby increasing the expression of cyclin-dependent kinase inhibitor 1C (CDKN1C) to promote cell survival [74]. Moreover, Snail could promote tumor invasion and metastasis under the regulation of METTL3. Mechanistically, the mobility of NPC cells could be enhanced upregulated Snail through the METTL3-m^6^A-IGF2BP2-dependent mechanism [77].

Consistent with NPC, overexpression of METTL3 is associated with tumorigenesis of OSCC. Liu et al. revealed that METTL3 could promote OSCC growth and metastasis through the METTL3-m^6^A-IGF2BP1-BMI1 axis [78]. In addition, METTL3 strengthened the stability of MYC in a METTL3-m^6^A-YTHDF1-mediated manner, thereby stimulating tumor progression [79]. Taken together, METTL3 could play the pro-oncogenic role in OSCC.

#### Lung cancer

METTL3 has been previously identified as a potential target for the treatment of NSCLC. The aberrant expression of METTL3 contributes to the tumorigenesis of NSCLC in multiple ways. METTL3-mediated m^6^A modification potentiated translation of YAP mRNA in a METTL3-m^6^A-YTHDF3-dependent manner, subsequently promoting the generation of cancer stem cells [36]. METTL3 installed m^6^A deposition on lncRNA ABHD11-AS1 and then enhanced aerobic glycolysis to propel tumor progression [80]. Additionally, the functional activation of METTL3 could promote rapid tumor growth and EMT, which dependent of the activation of PI3K/AKT pathways and the overexpression of EZH2 [38, 80]. In particular, elimination of miR-143-3p and vasohibin-1 (VASH1) induced by METTL3 resulted in enhanced brain metastasis [81]. Moreover, METTL3 could positively mediate the autophagy related pathway and further induce gefitinib resistance, indicating the potential role of METTL3 in the treatment of NSCLC [82].

In addition to NSCLC, dataset analysis revealed an upregulated METTL3 in lung adenocarcinoma (LUAD), which was correlated with poor prognosis of patients [83]. Although METTL3 is regarded as a potential biomarker of LUAD, the specific mechanisms remain to be further explored.

### Urological tumors

#### Renal cell carcinoma

The frequent alteration of METTL3 was reported in clear cell renal cell carcinoma (ccRCC), implying that METTL3 had potential predictive values in ccRCC [84]. Moreover, the close connection between METTL3 and critical biological processes was newly identified, including EMT, oxygen homeostasis, leukocyte migration, and so on [85, 86]. Since the underlying mechanisms are insufficient so far, it is necessary to conduct functional studies to explore the underlying mechanisms of METTL3 in ccRCC.

#### BC

Higher METTL3 is parallel with poor prognosis of patients with BC, suggesting the prognostic value and tumor promoting effect of METTL3 in BC [87]. It was demonstrated that METTL3 promoted rapid tumor growth, aggressive invasion, and self-renewal maintenance through different mechanisms. Abundant m^6^A deposition on the 3’UTR of CUB domain containing protein 1 (CDCP1) mRNA stimulated cell proliferation and transformation through the METTL3-m^6^A-YTHDF1 axis both in vitro and in vivo [88]. Similarly, the m^6^A modification within the 3’UTR of adhesive molecule integrin alpha-6 (ITGA6) mRNA permitted ITGA6 expression in an YTHDF1/3-dependent manner, thereby modulating the aggressive phenotype of BC [89]. In addition, METTL3 positively regulated the expression of endogenous AF4/FMR2 family member 4 (AFF4). The rapid tumor growth and aggressive invasion were ascribed to the AFF4/NF-κB/MYC pathway induced by METTL3, while self-renewal maintenance of BC stem cells was performed by the METTL3-AFF4-SOX2 axis [90, 91]. Tumor suppressor genes, such as SET domain containing 7 (SETD7) and Kruppel-like factor 4 (KLF4), are rapidly degraded under the regulation of METTL3 [92]. Furthermore, maturation of pri-miR221/222 associated with PTEN inhibition was conducted by METTL3, leading to poor prognosis in BC patients [34].

#### Prostate cancer (PCa)

METTL3 acts as an oncogene in PCa by promoting the pathogenesis and metastasis of tumor [93]. Mechanistically, METTL3 distinctly enhanced the expression of MYC, leading to the development and progression of PCa [94]. Besides, the METTL3-lymphoid enhancer-binding factor 1 (LEF1) axis activated the Wnt pathway in a METTL3-m^6^A-IGF2BP2-dependent manner, thereby promoting cell proliferation and migratory ability [95]. In addition, depletion of METTL3 disrupted the proliferation and immortality of tumor cells by inhibiting GLI1 in the sonic hedgehog (SHH) pathway [96]. Apart from tumor growth, bone metastasis was positively correlated with higher level of METTL3. Activation of human antigen R (HuR) induced by METTL3 resulted in the stability of integrin β1 (ITGB1) mRNA, thereby potentiating bone metastasis of PCa [97].

### Neurological tumors

#### Glioblastoma (GBM)

m^6^A modification is definitively involved in the tumorigenesis of GBM, but the roles of METTL3 are controversial. Methylation enrichment analysis revealed that m^6^A deposition was usually concentrated in the transcripts mediating cell growth, self-renewal and metabolic regulation pathways [98]. Mechanistically, upregulated METTL3 maintained the activation of glioblastoma stem cell (GSC) through regulating the RNA editing enzyme and the YTHDF2-mediated RNA decay [99]. In addition, METTL3 also mediated tumorigenesis of GBM independent of the methylase catalysis. Direct interaction between METTL3 and histone-mediated modification elevated the translation of oncogenes, including SOX2, spalt-like transcription factor 2 (SALL2), oligodendrocyte lineage transcription factor 2 (OLIG2) and POU class 3 homeobox 2 (POU3F2) [99]. On the other hand, METTL3 enhanced resistance to γ-irradiation by regulating m^6^A modification of SOX2, indicating the important role of METTL3 in therapeutic resistance [100].

A negative correlation between GSC and m^6^A modification was recently illustrated. Depletion of METTL3/14 in turn enhanced the cell proliferation and self-renewal ability, thereby strengthening the tumorigenic properties of GSC [101]. METTL3 could also impair the proliferation and mobility of glioma cells, indicating the dual role of METTL3 in GBM [102].

### Gynecologic tumors

#### Breast cancer

Previous studies reported that METTL3 was able to promote breast cancer cell proliferation by regulating the expression of BCL-2, hepatitis B X-interacting protein (HBXIP) and p21 through m^6^A [37, 103, 104]. Therapeutic resistance of breast cancer was also dependent on the m^6^A-based epitranscriptomic mechanism. Adriamycin resistance derived from METTL3 induced the maturation of pri-miRNA-221-3p [105], while tamoxifen resistance arose from METTL3-mediated overexpression of adenylate kinase 4 (AK4) [106]. Conversely, poor prognosis of the triple-negative breast cancer (TNBC) was associated with lower expression of METTL3, suggesting the tumor suppressing role of METTL3 [107]. Mechanistically, METTL3 inhibited the mobility of TNBC cells and adhesion to the cell extracellular matrix (ECM) by increasing m^6^A modification of collagen type III alpha Mechanistically, METTL3 inhibited the mobility of TNBC cells and adhesion to the cell extracellular matrix (ECM) by increasing m^6^A modification of collagen type III alpha 1 chain (COL3A1) [107]. Taken together, the differential functions of METTL3 were assessed in breast cancer, and various functions of METTL3 warrant further verification.

#### Ovarian cancer and endometrial cancer

METTL3 has been reported to promote tumor progression of ovarian cancer. METTL3 induced m^6^A modification in the transcripts of target genes in endometrioid epithelial ovarian cancer, including eIF3c, AXL, colony stimulating factor 1 (CSF-1), frizzled class receptor 10 (FZD10) and so on [108]. Upregulated AXL induced by methylation specifically promoted EMT to accelerate tumor progression [109]. METTL3 also participated in the regulation of oncogenic pathways in ovarian cancer. METTL3 downregulated the BCL-2-related apoptotic pathway to resist the apoptosis of ovarian cancer cells [110]. In addition, METTL3 mediated the activation of the AKT pathway via facilitating the maturation of miR-126-5p and further enhanced inhibition of PTEN, which was targeted by miR-126-5p [111].

Compared with ovarian cancer, METTL3 plays as a tumor suppressor in the pathogenesis of endometrial cancer. Mechanistically, reduced METTL3 could lead to activation of the AKT pathway, which promoted rapid proliferation of endometrial cancer cells [112].

#### Cervical cancer (CC)

METTL3 is identified as an independent prognostic factor in CC due to its distinct correlation with tumor progression and poor survival of patients [113, 114]. Increased METTL3 could bring to the rapid growth of CC through different mechanisms. Overexpression of METTL3 stabilized RAB2B mRNA to enhance cell proliferation in an IGF2BP3-dependent manner [115]. In addition, METTL3 was involved in m^6^A-regulated glycolysis, which was one of the critical hallmarks of tumor growth. METTL3 enhanced the stability of pyruvate dehydrogenase kinase 4 (PDK4) and HK2 mRNAs in a METTL3-m^6^A-YTHDF1-dependent manner, ultimately promoting tumor growth and chemoresistance [116].

### Hematological malignancies

#### Acute myeloid leukemia (AML)

AML is an aggressive hematological malignancy (HM) characterized by various genetic abnormalities and epigenetic dysregulation [117, 118]. Compared with normal progenitor cells, METTL3 was more abundant in AML cells, coupled with declined cell differentiation and apoptosis both in vitro and in vivo [119, 120]. Mechanistically, increased expression of METTL3 promoted the translation of MYC, BCL-2 and PTEN mRNAs, while depressing the differentiation-promoting effect of AKT [119].

#### Other types of hematological malignancies

Recent studies demonstrate that METTL3 participates in the development and progression of B-cell-derived hematological malignancies. Aberrant expression of METTL3 in acute B lymphoblastic leukemia (B-ALL) was profiled recently. Low expression of METTL3 was found in the ETV6/RUNX1 (E/R)-positive cohort and associated with high recurrence rate [121]. Compared with B-ALL, upregulated METTL3 was identified in B cell lymphoma. In contrast to ALL, m^6^A modification was enriched in the regulation pathways of cell division and RNA metabolism but also referred to favorable survival of mantle cell lymphoma (MCL) [122]. In addition, elevation of METTL3 in diffuse large B-cell lymphoma (DLBCL) promoted the expression of pigment epithelium-derived factor (PEDF) transcripts, thereby activating the Wnt pathway to accelerate cell proliferation [123]. Since particular mechanism of METTL3 is not yet sufficient at present, further studies on METTL3 in HM still needed.

### Head and neck squamous cell carcinoma and thyroid carcinoma

Emerging studies have demonstrated the pivotal role of METTL3 in head and neck squamous cell carcinoma (HNSCC) and thyroid carcinoma (TC). Dataset analysis revealed higher expression level of METTL3 in HNSCC, which was associated with poor OS and advanced tumor grade [124]. Similarly, METTL3 was highly expressed and closely associated with poor prognosis of TC [125]. Mechanistically, METTL3 regulated the expression of the HNF1 homeobox A (HNF1A) in a METTL3-m^6^A-IGF2BP2-dependent manner, eventually enhancing the migratory ability of tumor cells and activating the Wnt pathway [125].

## Targeting METTL3 in antitumor therapy

Based on the diverse functions of METTL3, targeting METTL3 may bring a novel perspective for individualized therapy of cancer. Meanwhile, the development of METTL3 inhibitors is feasible depending on structural and functional features. The functional domain of METTL3 can be considered as the target of inhibitors [126]. In particular, the cofactor S-adenosyl-L-methionine (SAM) in METTL3 was responsible for methyl group transfer, in which the competitive binding of small molecular complexes could effectively reduce the activity of methyltransferase [127]. From another perspective, METTL3 was commonly related to drug resistance in tumors. Chemoresistance induced by METTL3 were detected in several kinds of cancers [41, 66, 82, 105], indicating that functional inhibition of METTL3 might restore the chemosensitivity of tumor cells [127]. In addition, depletion of METTL3/14 could strengthen the therapeutic effect of anti-PD-1 therapy through activating the IFN pathway [68]. Therefore, targeting METTL3 may be regarded as a promising approach of tumor targeted therapy.

## Discussion

**Fig. 2:**
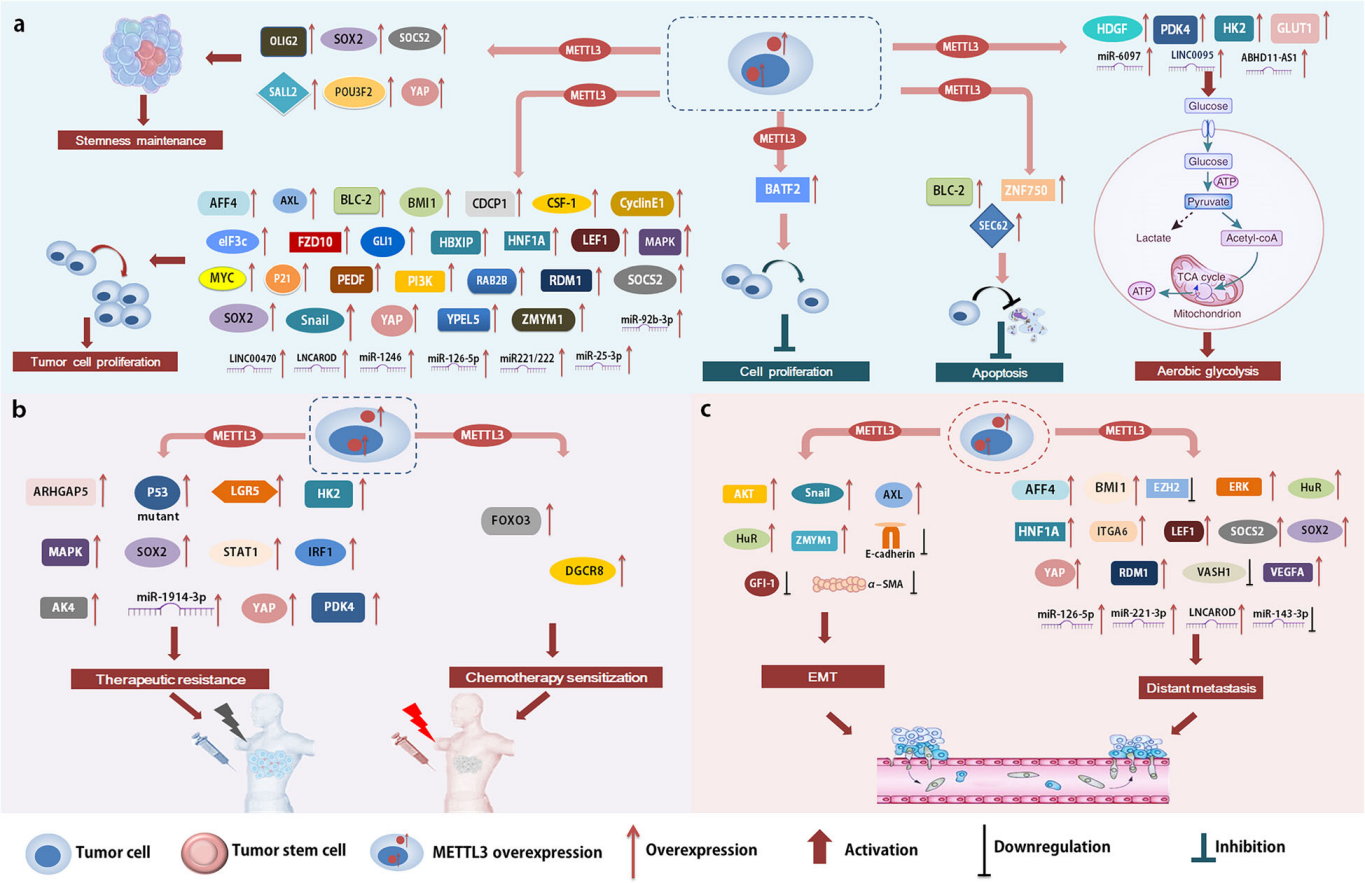
METTL3 regulates tumorigenesis and tumor progression by targeting downstream substrates. METTL3 is involved in many cellular processes, including tumor growth (**a**), therapeutic response (**b**), and metastasis (**c**)

Multiple functions of METTL3-mediated m^6^A modification have been determined in the pathogenesis and progression of tumors (Table 1), which is realized through regulating the expression and function of target genes (Fig. 2). Given the tumor-promoting effects of METTL3, targeting METTL3 brings a bright future for tumor targeted therapy. Nevertheless, more investigations are still required to explore the novel functions of METTL3.

**Table 1:** Multiple functions of METTL3 in human cancer

Cancer type	Expression	Role	Targets	Biological functions and underlying mechanisms	Refs
GC	Up	Oncogene	MYC	Promotes tumor progression by enhancing MYC expression	[44, 45]
			SEC62	Promotes anti-apoptosis by depressing the apoptosis pathway	[46]
			HDGF	Actives aerobic glycolysis by GLUT4 and ENO2 to promote tumor growth	[48]
			LncRNA LINC00470	Potentiates tumor growth by functional inhibition of PTEN	[40]
			ZMYM1	Promotes EMT by inhibiting E-cadherin expression	[49]
			GIF-1 and α-SMA	Enhances cell mobility and instant metastasis	[43]
			ARHGAP5	Enhances chemoradioresistance by stabilizing ARHGAP5 mRNA	[41]
		Suppressor	BATF2	Suppresses tumor progress by inhibiting the ERK pathway	[50]
			DGCR8	Enhances chemosensitivity to mTOR inhibitor	[51]
HCC	Up	Oncogene	SOCS2	Enhances cell proliferation, migration and stemness maintenance	[54]
			RDM1	Activates the Ras/Raf/ERK pathway to promote tumor progress	[55]
			Snail	Preserves oncogenic properties by up-regulating Snail	[56]
			miR-6079 and LINC00958	Enhances aerobic glycolysis by activating the mTOR pathway	[57, 58]
		Suppressor	FOXO3	Promotes sorafenib sensitivity, inhibits angiogenesis and autophagy	[59]
Gallbladder cancer	Up	Oncogene	miR-92b-3p	Inhibits PTEN to promote tumor progression	[60]
CRC	Up	Oncogene	SOX2	Promotes self-renewal, cell growth and metastasis	[61]
			Cyclin E1	Enhances tumor growth	[62]
			miR-1246	Promotes tumor progression by activating the MAPK pathway	[35]
			SOCS2 and YPEL5	Promotes tumor progression	[31, 63]
			HK2 and GLUT1	Accelerates aerobic glycolysis to promote tumor growth	[64]
			P53 and LGR5	Induces chemotherapeutic resistance	[66, 67]
			STAT1 and IRF1	Enhances resistance to anti-PD-1 treatment by inhibiting the IFN pathway	[68]
PC	Up	Oncogene	miR-25-3p	Promotes tumor growth and metastasis by activating the PI3K/AKT pathway	[70]
			MAPK	Induces chemo- and radio-resistance	[71]
NPC	Up	Oncogene	ZNF750	Suppresses cell apoptosis by inhibiting the ZNF750-FGF14 pathway	[75]
			EZH2	Enhances metastasis by up-regulating CDKN1C	[74]
			Snail	Enhances metastasis by increasing the translation of Snail mRNA	[77]
OSCC	Up	Oncogene	BMI1	Promotes cell proliferation and metastasis	[78]
			MYC	Promotes tumor progression by increasing the expression of MYC	[79]
NSCLC	Up	Oncogene	YAP	Stimulates stem cell generation and promotes tumor progression	[36]
			lncRNA ABHD11-AS1	Enhances aerobic glycolysis to promote tumor progression	[80]
			PI3K	Accelerates tumor growth by activating the AKT pathway	[38]
			EZH2	Enhances tumor metastasis by inhibiting the expression of EZH2	[80]
			miR-143-3p and VASH1	Enhances brain metastasis by eliminating miR-143-3p and VASH1	[81]
			autophagy	Induces chemotherapeutic resistance	[82]
BC	Up	Oncogene	CDCP1	Stimulates cell proliferation and transformation by enhancing CDCP1 expression	[88]
			ITGA6	Enhances aggressive features of tumor cell by enhancing ITGA6 expression	[89]
			AFF4	Accelerates tumor growth and invasion by activating MYC	[90]
				Maintains self-renewal capability of stem cells by activating SOX2	[91]
			SETD7 and KLF4	Promotes tumor progression by inhibiting the expression of SETD7 and KLF4	[92]
			pri-miR221/222	Induces poor prognosis of BC by functional inhibition of PTEN	[34]
PCa	Up	Oncogene	MYC	Promotes tumor progression	[94]
			LEF1	Promotes cell proliferation and metastasis by activating the Wnt pathway	[95]
			GLI1	Enhances cell growth and survival by activating the SHH pathway	[96]
			HuR	Promotes bone metastasis by enhancing the expression of ITGB1	[97]
GBM	Up	Oncogene	SOX2, SALL2, OLIG2 and POU3F2	Elevates the translation of oncogenes to activate stem-like cell	[99]
			SOX2	Enhances γ-irradiation resistance by elevating SOX2 expression	[100]
Breast cancer	Up	Oncogene	BCL-2, HBXIP and p21	Promotes cell proliferation by enhancing the expression of oncogenes	[37, 103, 104]
			Pri-miRNA-221-3p	Induces adriamycin resistance by facilitating miRNA maturation	[105]
			AK4	Induces tamoxifen resistance	[106]
TNBC	Up	Suppressor	COL3A1	Inhibits cell mobility and ECM adhesion	[107]
Ovarian cancer	Up	Oncogene	eIF3c, CSF-1 and FZD10	Promotes tumor progression and poor prognosis	[108]
			AXL	Promotes tumor progression by increasing the translation of AXL	[109]
			BCL-2	Suppresses cell apoptosis by inhibiting BCL-2 related pathway	[110]
			miR-126-5p	Enhances cell growth, migration and invasion by inhibiting PTEN	[111]
Endometrial cancer	Up	Suppressor	AKT regulators	Inhibits proliferation and tumorigenicity by inhibiting the AKT pathway	[112]
CC	Up	Oncogene	RAB2B	Enhances cell proliferation by increasing RAB2B expression	[115]
			PDK4 and HK2	Enhances aerobic glycolysis to promote tumor growth and chemoresistance	[116]
AML	Up	Oncogene	MYC and BCL-2	Promotes cell growth and anti-apoptosis	[119]
			PTEN	Suppresses cell differentiation by inhibiting the AKT pathway	
DLBCL	Up	Oncogene	PEDF	Promotes cell proliferation by activating the Wnt pathway	[123]
HNSCC	Up	Oncogene	lncRNA LNCAROD	Promotes cell proliferation and metastasis	[124]
TC	Up	Oncogene	HNF1A	Enhances migration by activating the Wnt pathway	[125]

Previous studies suggest that METTL3 usually regulates target genes in an m^6^A-dependent manner. In addition, biological functions of METTL3 can be independent of the methylase catalytic activity. For instance, the direct combination of METTL3 and ribosomes promoted the interaction between METTL3 and translation initiation, thereby enhancing the translation of mRNA and promoting tumor progression of lung cancer [128]. In addition, METTL3 also exhibited co-transcriptional interactions via regulating histone modification [129], indicating that METTL3 could interact with other types of epigenetic modification. On the other hand, mechanistic studies on METTL3 remain insufficient. For example, enrichment analysis uncovered the importance of METTL3 in glucose and lipid metabolism [130], but the specific mechanisms of METTL3 in tumor lipid metabolism were in infancy. Furthermore, mechanistic studies of METTL3 in some types of cancer, such as ccRCC, HNSCC and TC, were incomplete. Taken together, underlying mechanisms of METTL3 warrant further investigation.

In addition to exploring the novel functions of METTL3, fundamental researches also aim to achieve clinical transformation. Based on the tumor-promoting effect of METTL3, targeting METTL3 is expected to be an effective strategy for anti-tumor therapy. Functional inhibition of METTL3 was found to restore chemosensitivity of tumor cells in vitro, implying that inhibition of METTL3 might have potential value in vivo. In other words, application of METTL3 inhibitor is possible to produce anti-tumor effect and provide a solution for patients with refractory features. Investigations of METTL3-targeted therapies are an irresistible trend.

## Conclusions

The importance of METTL3 in tumor progression has been broadly identified in human cancer. METTL3 mainly promotes cell proliferation, invasion, migration, metabolic reprogramming, and drug resistance in cancer. Further investigations on the underling mechanisms and targeted inhibitors of METTL3 are of great significance for deeper understanding of the relationship between m^6^A modification and human cancer.

## Data Availability

Not applicable.

## Abbreviations

ac^4^C: N4-acetylcytidine
AFF4: AF4/FMR2 family member 4
AKT: Protein kinase B
AK4: Adenylate kinase 4
AML: Acute myeloid leukemia
ARHGAP5: Rho GTPase activating protein 5
B-ALL: Acute B lymphoblastic leukemia
BATF2: Basic leucine zipper ATF-like transcription factor 2
BC: Bladder cancer
CBLL1: Cbl proto-oncogene like 1
CC: Cervical cancer
ccRCC: Clear cell renal cell carcinoma
CDCP1: CUB domain containing protein 1
CDKN1C: Cyclin-dependent kinase inhibitor 1C
CDS: Coding sequence
COL3A1: Collagen type III alpha 1 chain
CRC: Colorectal cancer
CSF-1: Colony stimulating factor 1
DLBCL: Diffuse large B-cell lymphoma
ECM: Cell-extracellular matrix
eIF3h: Eukaryotic translation initiation factor 3 subunit h
EMT: Epithelial mesenchymal transition
ENO2: Enolase 2
E/R: ETV6/RUNX1
ERK: Extracellular-regulated kinase
EZH2: Enhancer of zeste homolog 2
FGF14: Fibroblast growth factor 14
FOXO3: Forkhead box O3
FZD10: Frizzled class receptor 10
GBM: Glioblastoma
GC: Gastric cancer
GFI-1: Growth factor independent 1
GLUT4: Solute carrier family 2 member 4
GSC: Glioblastoma stem cell
HBXIP: Hepatitis B X-interacting protein
HCC: Hepatocellular carcinoma
HDGF: Hepatoma-derived growth factor
HK2: Hexokinase 2
HM: Hematological malignancy
hm^5^C: 5-hydroxymethylcytidine
HNF1A: HNF1 homeobox A
HNSCC: Head and neck squamous cell carcinoma
HuR: Human antigen R
HUVECs: Human umbilical vein endothelial cells
IFN-γ: Interferon-γ
IRF1: Interferon regulatory factor 1
ITGA6: Integrin alpha-6
ITGB1: Integrin β1
KLF4: Kruppel-like factor 4
LEF1: Lymphoid enhancer-binding factor 1
LGR5: Leucine-rich repeat-containing G protein-coupled receptor 5
lncRNA: Long non-coding RNA
LUAD: Lung adenocarcinoma
m^6^A: N6-adenosine methylation
MAPK: Mitogen-activated protein kinase
m^5^C: Cytosine hydroxylation
MCL: Mantle cell lymphoma
METTL3: Methyltransferase-like 3
miRNA: MicroRNA
mRNA: Messenger RNA
MTC: N6-methyltransferase complex
mTOR: Mammalian target of the rapamycin
NPC: Nasopharyngeal carcinoma
NSCLC: Non-small cell lung cancer
OLIG2: Oligodendrocyte lineage transcription factor 2
OSCC: Oral squamous cell carcinoma
PC: Pancreatic cancer
PCa: Prostate cancer
PDK4: Pyruvate dehydrogenase kinase 4
PEDF: Pigment epithelium-derived factor
PI3K: Phosphatidylinositol 3'-kinase
Pri-miRNA: Primary miRNA
POU3F2: POU class 3 homeobox 2
PTEN: Phosphate and tension homology deleted on chromsome ten
RBM15: RNA-binding motif protein 15
RDM1: RAD52 motif 1
SALL2: Spalt-like transcription factor 2
SAM: S-adenosyl-L-methionine
SETD7: SET domain containing 7
SHH: Sonic hedgehog
SOCS2: Suppressor of cytokine signaling 2
SOX2: SRY-box 2
SPRED2: Sprouty-related EVH1 domain protein 2
STAT1: Signal transducer and activator of transcription 1
TC: Thyroid carcinoma
TNBC: Triple-negative breast cancer
VASH1: Vasohibin-1
WTAP: Wilms tumor 1-associated protein
YPEL5: Yippee-like 5
ZC3H13: Zinc finger CCCH-type containing 13
ZMYM1: Zinc finger MYM-type containing 1
ZNF750: Zinc finger protein 750
α-SMA: α-smooth muscle actin
